# Compensatory growth responses to food restriction in the Chinese three-keeled pond turtle, *Chinemys reevesii*

**DOI:** 10.1186/2193-1801-3-687

**Published:** 2014-11-24

**Authors:** Chunxia Xu, Wei Xu, Hongliang Lu

**Affiliations:** Hangzhou Key Laboratory for Animal Adaptation and Evolution, School of Life and Environmental Sciences, Hangzhou Normal University, Hangzhou, 310036 People’s Republic of China

**Keywords:** *Chinemys reevesii*, Compensatory growth, Food consumption, Carcass composition

## Abstract

Juvenile Chinese three-keeled pond turtles (*Chinemys reevesii*) were subjected to one of four different feeding regimens: *ad libitum* (AL), restricted (R), *ad libitum*-restricted (AL-R), or restricted-*ad libitum* (R-AL) for 13 weeks, to assess the compensatory growth (CG) response to food restriction and subsequent re-alimentation. After switching to *ad libitum* feeding, the turtles in R-AL group ate more food and grew faster than those in other groups. At the end of the trial, R-AL turtles achieved the comparable body weight as AL turtles, indicating that a complete CG response occurred. Cumulative food consumption over the entire period did not differ between R-AL turtles and AL turtles. Experimental treatment affected carcass composition. Carcass lipid content of AL turtles was greater than that of R and AL-R turtles, with R-AL turtles in between. Carcass protein content of R-AL turtles was slightly greater than that of other groups without statistical differences. Stored lipids might be consumed firstly when animals underwent food restriction. Our results reconfirmed the CG of *C. reevesii* after food restriction. However, it is still difficult to achieve a reduction in the cost of farm-raised turtle production by adopting a restricted–satiation feeding protocol.

## Background

For many centuries, turtles have been used as food, pets and in traditional medicine in different regions of the world (Fordhama et al. 2007; Mutalib et al. 2013). However, long-term over-exploitation of wild turtles is currently threatening their survival (Fong et al. 2007; Buhlmann et al. 2009). In the past few decades, a number of turtle species have been artificially cultured in an effort to satisfy the increasing demand for turtles in countries such as China. In commercial aquaculture, greater growth rate means a shortening of the culture cycle of farm-raised animals, of which could effectively reduce food consumption and production costs, therefore, finding the proper husbandry strategies to increase the growth rates of aquatic animals is very critical for farmers.

Compensatory growth (CG), the phase of accelerated growth following a period of feed restriction, has been observed in various organisms from mollusks to mammals (Wilson and Osbourn 1960; Fermin 2002; Vonesh and Bolker 2005; Wei et al. 2008; Roark et al. 2009). In many cases, food-restricted or food-deprived animals can eventually achieve the same or even a greater body size upon return to favorable food conditions, compared with those that have not experienced food restriction (Ali et al. 2003; Jobling 2010; Won and Borski 2013). Accordingly, it may be possible to exploit the principle of CG to improve the growth rates of farm-raised animals. This has previously been demonstrated in certain fish species (Jobling et al. 1994; Hayward et al. 1997; Chatakondi and Yant 2001). For example, juvenile hybrid sunfish (*Lepomis macrochirus* × *L. gibbosus*) that undergo repeating cycles of deprivation and re-feeding grow significantly faster and achieve a greater size at the same age than controls that are fed to satiation daily (Hayward et al. 1997). Among cultured aquatic species, studies addressing CG have focused mainly on fish (e.g., Hayward et al. 2000; Oh and Noh 2006; Srijila et al. 2014). Although an evident compensatory response to food deprivation has been found in some cultured freshwater turtles (Xie and Niu 2007; Wang et al. 2011; Xie et al. 2012), it remains unclear whether the growth rate of these turtles can be improved by exploiting the CG response.

The Chinese three-keeled pond turtle, *Chinemys reevesii*, is a species that is widely distributed in eastern Asia from Japan to southern China. This species is one of the most commercially important turtles for aquaculture and is widely cultured in China (Cheung and Dudgeon 2006; Du et al. 2007). A CG response to complete food deprivation in *C. reevesii* was demonstrated in a previous study, in which deprived turtles were refed to satiation for 4 weeks, but did not achieve the same size as controls (Wang et al. 2011). However, the magnitude of compensatory growth may depend on the developmental stage of the animals, and the intensity and duration of feed restriction (Ali et al. 2003). Seasonal fluctuations in food availability are ubiquitous in the natural environment and wild animals often undergo intermittent, partial food deprivation rather than prolonged, complete food deprivation. In fact, the growth responses of cultured turtles to limited food availability remain largely unstudied. More detailed information is necessary in order to determine whether CG can be used to improve the growth rate of *C. reevesii.* In the present study, we assessed the compensatory responses of juvenile *C. reevesii* to food restriction followed by increased food availability, thereby providing useful information for turtle husbandry practices.

## Materials and methods

### Animal collection and maintenance

A total of 62 juvenile turtles, about 2 months after hatching, were obtained from a private hatchery in Haining (Zhejiang, eastern China), and transferred to our laboratory at Hangzhou Normal University, where they were weighed and measured for carapace length and width. The turtles were housed individually in 30 × 20 × 25 cm^3^ aquaria that contained water to a depth of 5 cm. Aquaria were kept in a temperature-controlled room at 30°C under a 12 h light:12 h dark cycle. Pieces of tiles were placed in the aquaria to provide shelters for the turtles, and the water was replaced daily. Turtles were fed a commercially available diet (food composition: 10% water, 47% crude proteins, 8% lipids and 7% carbohydrates) daily, and the food pellets that remained in each aquarium were counted every afternoon. Approximate food consumption was calculated as the difference between the mass of food offered and the estimated mass of food remaining (the number of remaining food pellets × the average mass per pellet). Turtles were weighed weekly.

After 2 weeks of acclimation to the laboratory, turtles were randomly assigned to one of four treatment groups following Roark et al. ( 2009): *ad libitum* (AL; fed *ad libitum* for 13 weeks, *N* = 13), restricted (R; fed ~25% of initial *ad libitum* intake for 13 weeks, *N* = 13), *ad libitum*-restricted (AL-R; fed *ad libitum* for 6 weeks and then food-restricted for 7 weeks, *N* = 13), and restricted-*ad libitum* (R-AL; food-restricted for 6 weeks and then fed *ad libitum* for 7 weeks, *N* = 13). The remaining turtles (*N* = 10) were killed and hereafter are referred to as 0-week turtles.

### Carcass composition

After 13 weeks, all turtles were euthanized by freezing to -15°C for later determination of composition. Each turtle was separated into the carcass (including head, limbs, tail, carapace and plastron) and internal organs. The carcasses were dried to constant mass in an oven at 65°C, and then weighed to the nearest 0.1 mg on a Mettler Toledo balance (model AB135-S). The whole dried carcass was ground in a Wiley mill to a fine powder for subsequent analyses of the lipid and protein content. We extracted non-polar lipids from dried carcass samples in a Soxhlet apparatus for 5.5 h using absolute ether as solvent. The lipid content of each sample was determined by subtracting the lipid-free dry mass from the total sample dry mass. Nitrogen content was determined by the Kjeldahl method (AOAC 1984), and protein content was calculated by multiplying nitrogen content by 6.25.

### Data analysis

One turtle in R-AL group died during the experiment and the corresponding data were excluded from statistical analysis. The specific growth rate (SGR) and feed efficiency ratio (FER) were respectively calculated as SGR = (ln*W*_t_ - ln*W*_0_)/*T* × 100% and FER = (*W*_t_ - *W*_0_)/*C*_w_ × 100%, where *W*_0_ = initial wet body mass, *W*_t_ = final wet body mass, *T* = duration of experiment and *C*_w_ = wet mass of food consumed. Statistical analyses were performed using STATISTICA 6.0 (StatSoft Inc. OK, USA). We used linear regression, one-way analysis of variance (ANOVA), repeated measures ANOVA, multivariate analysis of variance (MANOVA) and Tukey’s *post hoc* test to analyze the corresponding data that met the assumptions for parametric analyses. Before conducting parametric analyses, all variables were tested for normality using the Kolmogorov-Smirnov test and for homogeneity of variances using Bartlett’s (at univariate level) or Box’s M (at multivariate level) test. Throughout the paper, values are presented as mean ± SE, and the significance level is set at *P* = 0.05.

## Results

**Figure 1:**
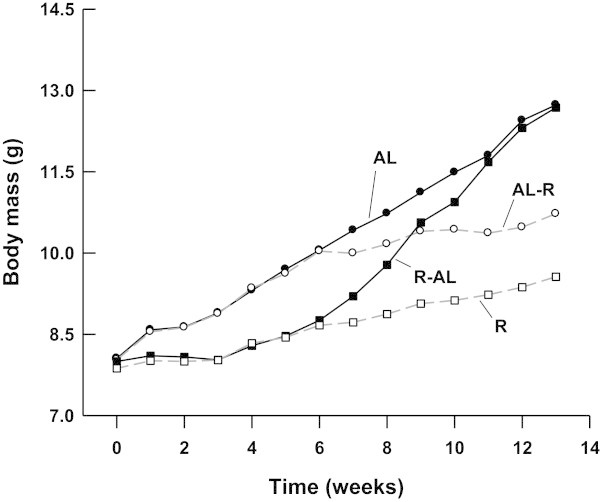
**Mean body mass of juvenile** ***Chinemys reevesii*** **fed** ***ad libitum*** **(AL), restricted (R),** ***ad libitum*** **-restricted (AL-R), or restricted-** ***ad libitum*** **(R-AL) diets.** Body mass was measured at the end of each week.

The body size (mass) of turtles did not differ among groups at week 0 (prior to the beginning of the experiment) (one-way ANOVA, *F*_4, 56_ = 0.14, *P* = 0.966), but significantly differed at week 6 (prior to the diet switch) (*F*_3, 47_ = 3.39, *P* = 0.026) and at week 13 (the end of the experiment) (*F*_3, 47_ = 7.72, *P* <0.001). Repeated measures ANOVA revealed significant effects of time (*F*_13, 611_ = 172.70, *P* <0.0001), treatment (*F*_3, 47_ = 3.09, *P* = 0.036), and a time × treatment interaction (*F*_39, 611_ = 12.05, *P* <0.0001) on body size throughout the trial. The mean body size of R-AL and R turtles was significantly smaller than that of AL and AL-R turtles at week 6. After *ad libitum* feeding for 7 weeks, mean body size of R-AL turtles was similar to that of AL turtles, and was significantly larger than that of R turtles (Figure 1).

**Figure 2:**
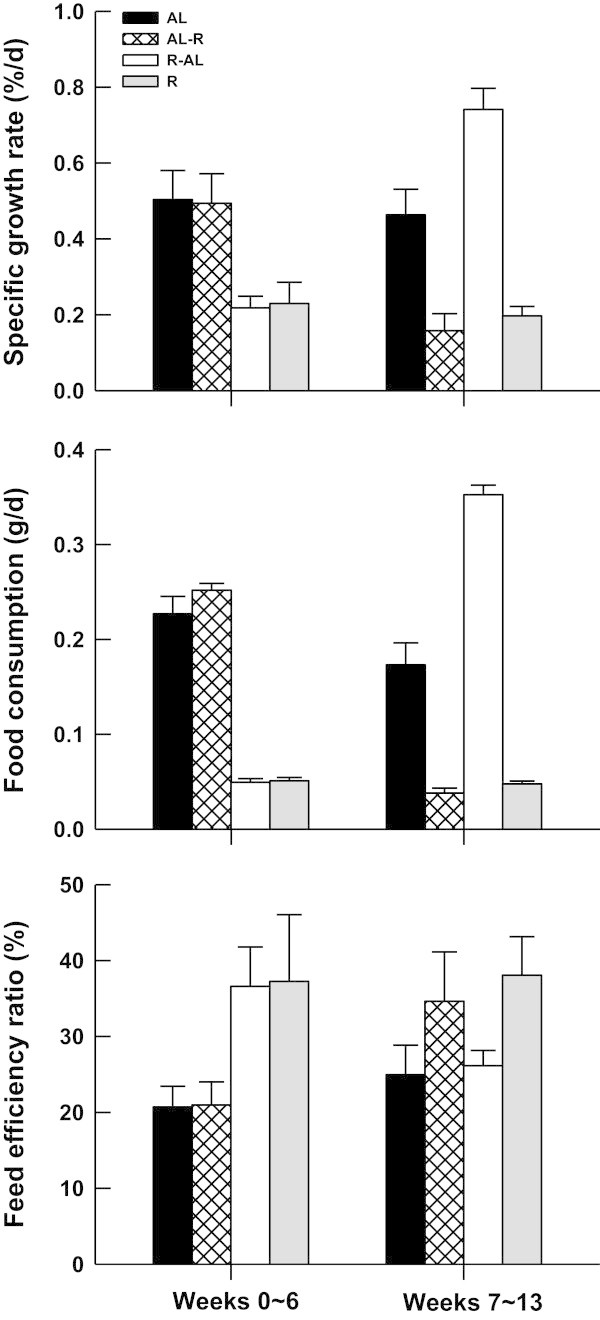
**Specific growth rate, food consumption, and feed efficiency ratio for juvenile** ***Chinemys reevesii*** **fed** ***ad libitum*** **(AL), restricted (R),** ***ad libitum*** **-restricted (AL-R), or restricted-** ***ad libitum*** **(R-AL) diets.** Data are expressed as mean + SE.

**Figure 3:**
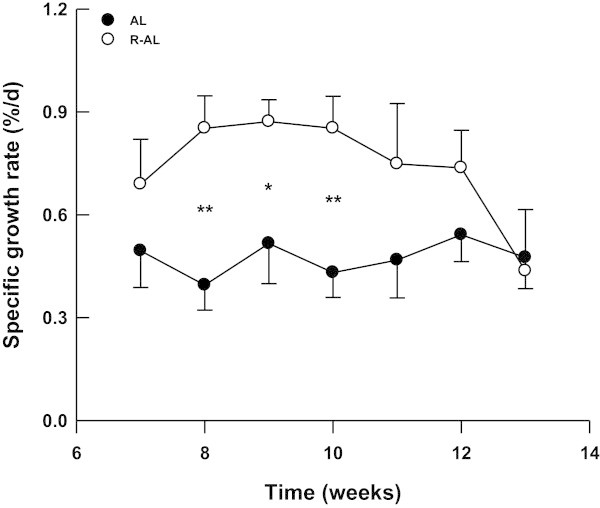
**Specific growth rate of** ***Chinemys reevesii*** **fed** ***ad libitum*** **(AL; mean - SE) and restricted-** ***ad libitum*** **(R-AL; mean + SE) diets in weeks 7–13.** The asterisks denote significant differences in specific growth rate between AL and R-AL turtles. *P <0.05, **P <0.01.

The potential influences of body size on the growth rate, food consumption and FER of the turtles were not found among treatments (linear regression, all *P* >0.05). Food-restricted turtles were had significantly lower food consumption (one-way ANOVA, *F*_3, 47_ = 128.09, *P* <0.0001, AL-R = AL > R = AL-R) and SGR (*F*_3, 47_ = 6.09, *P* <0.002, AL = AL-R > R = R-AL), but higher FER (*F*_3, 47_ = 2.86, *P* = 0.047) than *ad libitum*-fed turtles during weeks 0–6 (Figure 2). R-AL turtles consumed significantly more food (repeated measures ANOVA, *F*_1, 11_ = 884.16, *P* <0.0001) and exhibited a higher growth rate (*F*_1, 11_ = 89.41, *P* <0.0001) after the switch to *ad libitum* feeding, whereas AL-R turtles exhibited a lower growth rate (*F*_1, 12_ = 9.86, *P* <0.01) due to restricted feeding (*F*_1, 12_ = 1028.77, *P* <0.0001). Food consumption (one-way ANOVA, *F*_3, 47_ = 122.97, *P* <0.0001, R-AL > AL > R = AL-R) and SGR (*F*_3, 47_ = 27.76, *P* <0.0001, R-AL > AL > R = AL-R) also differed between groups during weeks 7–13, with R-AL turtles having a greater food intake and growing faster than turtles in other groups (Figure 2). Over the 13-week period, the cumulative food consumption of R-AL turtles was similar to that of AL turtles, but was significantly higher than those of AL-R and R turtles (one-way ANOVA, *F*_3, 47_ = 112.25, *P* <0.0001, AL = R-AL > AL-R = R). In order to assess CG capacity, we excluded the data from AL-R and R turtles, and compared R-AL turtles to AL turtles in weeks 7–13. After the switch to *ad libitum* feeding, the SGR of R-AL turtles was higher than that of AL turtles in the first few weeks, and gradually returned to the level of AL turtles in the last 3 weeks (Figure 3). The FER of turtles did not differ among groups during weeks 7–13 (one-way ANOVA, *F*_3, 47_ = 1.83, *P* = 0.154).

Experimental treatment affected carcass composition (MANOVA, Wilks’ *λ* = 0.44, *df* = 15, 119, *P* <0.01). Carcass wet and dry masses of AL and R-AL turtles were greater than that of R turtles. The water content of wet carcass did not differ among groups. The lipid content of dry carcass of AL turtles was greater than those of R and AL-R turtles, but did not differ from that of R-AL turtles. The protein content of R-AL was slightly greater than that of other groups, but the difference was not statistically significant (Table 1). Carcass wet and dry masses of 0-week turtles were significantly smaller than those of the experimental turtles. The lipid content of 0-week turtles was greater than those of AL-R and R turtles, but did not differ from those of AL and R-AL turtles (Table 1).

**Table 1:** **Carcass composition of juvenile** ***Chinemys reevesii*** **at 0- and 13-weeks**

Group	***N***	Body wet mass (g)	Dry mass (g)	Water content (%)	Lipid content (%)	Protein content (%)
0-week turtles	10	5.46 ± 0.18^c^	1.31 ± 0.05^c^	75.7 ± 1.4	11.2 ± 0.6^a^	7.8 ± 0.6
AL	13	9.82 ± 0.56^a^	2.39 ± 0.12^a^	75.5 ± 0.5	10.5 ± 0.6^a^	7.6 ± 0.4
AL-R	13	8.37 ± 0.54^ab^	1.86 ± 0.22^bc^	77.5 ± 2.7	8.2 ± 0.7^b^	8.2 ± 0.6
R-AL	12	9.70 ± 0.35^a^	2.22 ± 0.13^ab^	77.1 ± 1.1	9.9 ± 0.7^ab^	8.7 ± 0.3
R	13	7.36 ± 0.18^b^	1.68 ± 0.04^c^	77.1 ± 0.3	8.0 ± 0.5^b^	7.0 ± 0.4

Data are expressed as mean ± SE. Means with different lettered superscripts differ significantly (Tukey’s *post hoc* test, a > b > c).

## Discussion

Consistent with the results of Wang et al. ( 2011), an obvious CG response was observed in juvenile *C. reevesii* that experienced a period of feed restriction. Food-restricted turtles caught up in body size with those that had not undergone feed restriction, indicating a complete CG pattern in *C. reevesii*. However, Wang et al. ( 2011) reported a partial CG response in this turtle. The expression of CG responses can be affected by several factors, such as the size of the animals, the feeding regime, and the intensity and duration of feed restriction (Kim and Lovell 1995; Thompson et al. 2000; Ali et al. 2003). The differences between the results of these two studies may be due to differences in the experimental methods or the size of animals. Turtles in the Wang et al. ( 2011) study had relatively large size and were completely deprived of food for no more than 4 consecutive weeks, whereas R-AL turtles in the present study were fed a restricted ration for 6 weeks. The deprivation period should be long enough (more than 2 weeks) to evoke a detectable CG response, and a longer deprivation period seems to result in a more pronounced response in *C. reevesii* (Wang et al. 2011). The complete CG response we observed was probably a result of the relatively long period of food restriction. Therefore, the results in our study may not contradict those reported by Wang et al. ( 2011), but provide evidence that the intensity and duration of feed restriction affects the CG response of juvenile turtles. Moreover, the influence of feed restriction intensity on CG response may vary among species or populations. For example, restricted feeding induces an obvious CG response in *C. reevesii*, which has also been observed in two sea turtles, *Caretta caretta* and *Chelonia mydas* (Bjorndal et al. 2003; Roark et al. 2009). However, another freshwater turtle, *Pelodiscus sinensis*, exhibited only a partial CG response to complete food deprivation (Xie and Niu 2007).

CG can be attributed to starvation-induced physiological changes (Ali et al. 2003; Gurney et al. 2003; Won and Borski 2013). After the switch to *ad libitum* feeding, food consumption of R-AL turtles were significantly enhanced and greater than that of AL turtles, whereas the FER in R-AL turtles was lower than other groups without statistical differences. This suggests that an increase in food consumption (i.e., hyperphagia), rather than the efficiency of food conversion, may be the major cause for CG in *C. reevesii*. Enhancement of growth rates by increasing food consumption was also found in the previous study (Wang et al. 2011), and in *P. sinensis* (Xie and Niu 2007) and some fish species (Chatakondi and Yant 2001; Tian and Qin 2004). CG may also be achieved by improving food conversion and reducing metabolic rate (Ali et al. 2003; Roark et al. 2009). Completely food-deprived *C. reevesii* juveniles have lower metabolic rates than those fed to satiation (Lu and Wang 1993). The reduced metabolic rate of deprived turtles might persist for the first few days of refeeding period, and contributes to the CG response (Wang et al. 2011). The restricted ration used in this study may have exceeded basal maintenance costs because food-restricted turtles continued to grow during the restriction period. Whether the metabolic rate of food-restricted turtles is reduced during the periods of restriction and *ad libitum* feeding should be determined in future research.

The lipid content of dry carcasses appears to be related to the duration of feed restriction, with turtles that experienced a longer period of food restriction having lower carcass lipid content. However, R-AL turtles tended to have relatively high carcass protein content at the end of the experiment (Table 1). Our results suggest that stored lipids may be mobilized prior to the mobilization of proteins when animals undergo food restriction or deprivation. Such pattern was also exhibited in the previous study (Wang et al. 2011), and in *P. sinensis* (Xie and Niu 2007) and some fish species (Qian et al. 2000; Luo et al. 2009).

In summary, the present study confirmed that juvenile three-keeled pond turtles exhibit a complete CG response following a period of food restriction. However, the R-AL feeding regime that we used did not reduce the total food consumption of turtles over the entire experiment. Accordingly, this protocol is not recommended for aquaculture practices, because it fails to evoke an overcompensation of growth, and thus shorten the duration of culture cycles or reduce food consumption of farm-raised turtles.
